# *‘I’m proud of how far I’ve come. I’m just ready to work’*: mental health recovery narratives within the context of Australia’s Disability Employment Services

**DOI:** 10.1186/s12889-020-8452-z

**Published:** 2020-03-12

**Authors:** Alexandra Devine, Cathy Vaughan, Anne Kavanagh, Helen Dickinson, Sean Byars, Stefanie Dimov, Bill Gye, Lisa Brophy

**Affiliations:** 1 Centre for Health Equity, Melbourne School of Population and Global Health, University of Melbourne, Melbourne, Australia; 2 Public Service Research Group, School of Business, University of New South Wales, Canberra, Australia; 3 Community Mental Health Australia, Sydney, Australia; 4 Centre for Mental Health, Melbourne School of Population and Global Health, University of Melbourne, Melbourne, Australia; 5 Social Work and Social Policy, School of Allied Health, Human Services and Sport, La Trobe University, Melbourne, Australia

**Keywords:** Personal recovery, Mental illness, Psychosocial disability, Disability employment services

## Abstract

**Background:**

Employment is recognised as facilitating the personal and clinical recovery of people with psychosocial disability. Yet this group continue to experience considerable barriers to work, and, constitute a significant proportion of individuals engaged with Disability Employment Services (DES). Recognition of the role of recovery-oriented practice within DES remains limited, despite these approaches being widely promoted as best-practice within the field of mental health.

**Methods:**

The Improving Disability Employment Study (IDES) aims to gather evidence on factors influencing employment outcomes for Australians with disability. Descriptive analysis and linear regression of IDES survey data from 369 DES participants, alongside narrative analysis of data collected through 56 in-depth interviews with 30 DES participants with psychosocial disability, allowed us to explore factors influencing mental health, well-being and personal recovery within the context of DES.

**Results:**

Psychosocial disability was reported as the main disability by 48% of IDES respondents. These individuals had significantly lower scores on measures of mental health and well-being (44.9, 48.4 respectively, *p* ≤ 0.01), compared with respondents with other disability types (52.2, 54.3 p ≤ 0.01). Within this group, individuals currently employed had higher mental health and well-being scores than those not employed (47.5 vs 36.9, 55.5 vs 45.4 respectively, *p* ≤ 0.01). Building on these findings, our qualitative analysis identified five personal recovery narratives: 1) Recovery in spite of DES; 2) DES as a key actor in recovery; 3) DES playing a supporting role in fluctuating journeys of recovery; 4) Recovery undermined by DES; and, 5) Just surviving regardless of DES. Narratives were strongly influenced by participants’ mental health and employment status, alongside the relationship with their DES worker, and, participants’ perspectives on the effectiveness of services provided.

**Conclusion:**

These findings re-iterate the importance of work in supporting the mental health and well-being of people with psychosocial disability. Alongside access to secure and meaningful work, personal recovery was facilitated within the context of DES when frontline workers utilised approaches that align with recovery-orientated practices. However, these approaches were not consistently applied. Given the number of people with psychosocial disability moving through DES, encouraging greater consideration of recovery-oriented practice within DES and investment in building the capacity of frontline staff to utilise such practice is warranted.

## Introduction

People with psychosocial disability are a sub-group of people whose experiences of mental illness (e.g. depression, anxiety, schizophrenia), in interaction with the socio-cultural context (e.g. norms around mental illness, stigma, availability of supports and services) in which they live, have led to disabling experiences [1–3]. Socio-economic inequalities and poor health outcomes experienced by people with psychosocial disability are both causes and consequences of their poorer employment outcomes. Their labour force participation (29%) and unemployment rate (19%) in Australia for example, are poorer in comparison to people with other disabilities (53.4 and 10% respectively), and substantially lower than the general population (83.2 and 5%) [2, 4, 5].

As in many Anglophile countries, recovery-orientated practice is one of the key principles guiding Australia’s mental health system to support people with mental illness to ‘create and live a meaningful and contributing life in a community of choice, with or without the presence of mental illness’ [6] pg 69]. Personal recovery can be distinguished from clinical recovery in being less focused on symptom reduction and more focused on well-being and having a flourishing life. Recovery-orientated practice aims to deliver services that recognise the uniqueness of individuals, empowering them to make choices about what recovery means to them and how they want to engage with services and receive supports that facilitate their personal goals [7–10].

Employment is widely recognised as facilitating both personal and clinical recovery [11]. Many people with psychosocial disability, however, continue to experience vocational (disrupted education and work histories) and non-vocational (discrimination, limited social networks) barriers to employment and often require support to attain employment [12]. The Disability Employment Services (DES) program is the Australian Government’s specialised welfare program for people whose disability is assessed as their main barrier to employment [13]. Of the more than 265,000 current DES participants, 40.7% are reported to have a psychosocial disability as their primary condition [14], and, employment services were the most commonly reported non-residential service accessed by the 100,866 people with psychosocial disability receiving supports provided under Australia’s National Disability Agreement (NDA) during 2017–18 [15]. Recovery-orientated practice however is absent from Government documentation pertaining to DES, with its overarching objective stated as being ‘to improve the nation’s productive capacity by employment participation of people with disability, thereby fostering social inclusion’ [[16] pg 11].

This paper explores whether and how DES participants with psychosocial disability experience recovery. The paper begins by discussing the relationship between mental health and work, exploring conceptualisations of recovery and recovery-orientated practice. A description of the IDES quantitative and qualitative methods follow. Quantitative findings examining the mental health and well-being of IDES respondents and factors which influence these measures are presented. We then describe the recovery narratives emerging from narrative analysis of the qualitative interview data. Lastly, we integrate study findings to discuss how mental health, well-being and recovery are influenced by engagement with DES, work and systems-level challenges, and, discuss approaches for improving employment support and recovery outcomes for DES participants with psychosocial disability.

### Mental health and work

People with psychosocial disability often experience exclusion across various life domains, contributing to socio-economic disadvantage in education, housing, employment and social participation. This disadvantage in turn influences health. When health needs are not met, mental health conditions are often exacerbated, compounding barriers to employment [1, 12, 17].

Substantial evidence highlights the importance of work in facilitating both clinical and personal recovery [11, 18, 19]. Work supports an individual’s economic security and improves standard of living. This in turn facilitates access to factors that inherently support physical and mental health, such as housing, transport and recreation. Work helps to provide structure to people’s daily lives, as well as contributing to a person’s sense of self and social connectedness [20–22]. Previous research has also found work plays a central role in recovery by encouraging people with mental illness to develop strategies to manage their symptoms [23].

Mental health can be negatively impacted, however, if work is of poor psychosocial quality [24, 25], such as when people are exposed to hazardous conditions or when tasks do not match skills, interests, or remuneration. Workplace bullying and discrimination, or situations where people do not feel they have a sense of control over their work, can further undermine the mental health of workers [26–30]. Casualization of work within precarious labour markets or in contexts of economic recession has been described as an important contributor to poor mental health in otherwise well individuals, as well as more vulnerable population groups [31, 32].

### Personal recovery

The early conceptualisations of personal recovery were strongly influenced by constructs of empowerment, self-determination and choice, as well as (re) claiming rights to a safe, dignified and personally meaningful life within communities, whilst living with a mental illness [33–35]. To formalise a model of recovery, Leamy and colleagues conducted a systematic synthesis of personal recovery published within mental illness research [17, 33]. The subsequent CHIME Personal Recovery Model incorporates a number of constructs that are both relevant to the career pathways of people with psychosocial disability as well as helping to understand the impact of employment on an individual’s personal recovery [36].

CHIME identifies five key dimensions of recovery: 1) **Connectedness** including concepts of peer support, relationships, support from others and being part of the community; 2) **Hope and optimism** in the future including a belief in the possibility of recovery, motivation to change, hope-inspiring relationships, and, having dreams and aspirations; 3) **Identity** through (re) building or (re) defining positive senses of identity, and, overcoming stigma; 4) Finding **Meaning** and purpose in the lived experience of mental illness and developing meaningful life and social roles and goals; and 5) **Empowerment** through personal responsibility and having a sense of control over one’s life [17, 33].

The CHIME model emphasises the need to recognise and value all individuals as people with hopes, dreams, desires and capabilities, rather than focusing predominantly on health conditions, impairments and barriers [37]. This highlights the importance of support services (health and employment), as well as the broader community, having the expectation that people with psychosocial disability can recover and work. Positive expectations should therefore inform the ways that services support individuals to lead their own recovery, including enabling them to have choice and control in relation to employment [37, 38].

### Recovery-orientated practice and DES

Recovery-oriented practices are based on a person-first approach that recognise the uniqueness of individuals and that people are the experts in their own lives. They aim to instil the belief that recovery outcomes are both personal and possible, supporting individuals to develop and engage with social, recreational, occupational and vocational activities that are meaningful to them. Service providers listen and learn from individuals through the development of respectful and trusting relationships, and, ensure individuals are well-informed, supported and empowered to use information to make choices about how they engage with services and the supports they receive. Importantly, recovery-orientated practices strive to challenge discrimination and stigma wherever it exists [7, 8, 39, 40].

Recovery-orientated practices are not emphasised within the context of DES policies or contractual arrangements [DSS 16]. DES sits within the broader welfare system and the majority of participants engage with DES as part of increasingly punitive mutual obligations to remain in receipt of income support. Indeed, it has been argued the punitive welfare-to-work measures that are increasingly seen within Australia’s welfare system, not only make it difficult to support key recovery elements such as empowerment and choice and control, but can be harmful for people experiencing long-term unemployment and significant unaddressed barriers to work [41–44].

DES has been further criticised as not enabling evidence-based practices known to support people with psychosocial disability into work, highlighted by the poorer employment outcomes attained by these participants [45, 46]. Poorer outcomes have been attributed to the limited collaboration between mental health services, alongside the undersupply of qualified vocational rehabilitation specialists working within the DES sector, with staff often reported as having minimal experience and training in working with people with mental health conditions [47–50]. When interactions between people with psychosocial disability and support services are not positive, processes of recovery can be further undermined [51, 52].

There is, however, evidence that DES frontline staff can be supported to develop skills that are more effective at helping people with psychosocial disability achieve work outcomes [53]. Research by King and Waghorn [54] found more effective DES frontline staff utilise positive working alliances with job seekers; incorporate psychological interventions within employment supports; and, work with employers to address stigma related to employing people with psychosocial disability. These approaches align with recovery-orientated practices and demonstrate the potential of DES to better support recovery and employment outcomes. Our study aims to build on this evidence by further exploring factors influencing the mental health and well-being and personal recovery of DES participants.

## Methods

This mixed-methods study was embedded within the Improving Disability Employment Study (IDES). Implemented by the University of Melbourne in partnership with disability and employment services peak bodies and nine DES providers located across Australia, IDES aims to gather evidence on factors that influence sustainable employment outcomes for Australians with disability. IDES involves a prospective cohort survey of 369 DES participants. Alongside the survey, qualitative data was collected through 56 in-depth interviews with 30 DES participants with psychosocial disability to more deeply explore their lived experience and their engagement with DES and work. Participants of both the IDES survey and qualitative interviews were all 18 years or above with informed consent collected prior to each survey and interview. Ethics approvals were obtained from the University of Melbourne’s Human Research Ethics Committee (ID 1545810.1 & 1,750,133.1).

## Quantitative methods

The IDES survey was piloted in February 2018 with 32 DES participants recruited through DES partners. Wave 1 of the survey was implemented between April and December 2018 with 337 survey respondents conveniently recruited through DES partner frontline workers or via an email link sent to DES clients [55]. As the majority of items remained the same from the pilot to Wave 1, we have combined pilot and Wave 1 data for the purpose of this paper. Participants completed an online version of the interview or via Computer-assisted Telephone Interview (CATI). The survey took 30–45 min to complete and explored functioning, health and well-being, socio-economic conditions, and engagement with employment services and work [55]. Survey participants are invited to complete a follow-up survey ~ 12 months after the first (Wave 2). Wave 2 is currently in the field with data collection due to be completed in early 2020.

### Quantitative data variables and analysis

Demographic and socio-economic variables included age, gender, education, and ethnicity. Variables on employment, housing, transport and finances were adapted from the Australian Survey of Disability, Aging and Carers (SDAC) [5], Life Opportunities Survey [56], and the Household, Income and Labour Dynamics in Australia survey (HILDA) [57] with some items also developed by the research team [55]. Mental health was measured using the five-item Mental Health Inventory (MHI-5), a subscale of the Short form-36 (SF-36) general health measure. The MHI-5 has been validated as a screening tool to detect symptoms of anxiety, depression, behaviour control, positive affect and general distress in the past 4 weeks [58]. Our analysis used a generated continuous MHI-5 total score (scale 1 to 100), with higher scores representing better mental health.

The seven-item Personal Wellbeing Index (PWI) was included as a validated measure of subjective well-being. The PWI items elicit respondent satisfaction across the domains of standard of living, health, achieving in life, relationships, community connectedness and future security. The PWI total scores corresponding to a continuous scale (1 to 100) were generated based on previously described frameworks [59, 60]. PWI items also correspond to various components across the CHIME recovery framework.

IDES respondents were defined as having a psychosocial disability through self-report (i.e. reported their main disability was psychological) and responses to the Washington Group (WG) Short and Extended Sets of questions. The WG on Disability Statistics designed these Sets to identify people at risk of disability through nationally-based surveys. The Extended Set items included pain, fatigue and affect items (anxiety and depression) with responses measuring frequency and severity of symptoms [61]. If a person did not self-report a specific disability they were assigned to the psychosocial disability group if their main reported difficulties across the Extended Set included daily or weekly anxiety or depression with the level reported as *‘a lot’* or ‘*somewhere in between a little and a lot’*, and, their responses to the Short Set of questions indicated no other or less difficulties in other domains (e.g. vision, hearing, mobility).

IDES survey data was entered into Stata 15 for analysis [62]. Descriptive analysis was undertaken to identify demographic and socio-economic characteristics (age, gender, education), and experiences in the labour market (access to paid work, factors impacting on access to work, discrimination) and differences between participants with psychosocial disability and participants with other types of impairments (i.e. participants with physical, sensory, cognitive or multiple impairments who were grouped together for the purpose of this analysis). Adjusting for age, gender and education (dichotomised by completed secondary school or did not complete secondary school), linear regression modelling using the MHI-5 and PWI as outcome variables were used to examine the associations between mental health and well-being and a range of exposure variables (engagement with DES and the labour market, discrimination, housing insecurity) in participants with a psychosocial disability. Findings helped inform the qualitative analysis and were used to triangulate narratives.

### Qualitative methods

Qualitative interview participants were recruited across two cohorts, one prior to and one after the DES program underwent reform in July 2018. Participants were recruited: 1) through the IDES survey, or 2) directly through DES partners. In the first method of recruitment, potential participants recruited through the IDES survey included respondents who: 1) gave consent as part of the survey to be contacted for follow-up interviews, and 2) identified through self-report within the survey as having a psychosocial disability. Eligible potential participants were contacted by the lead author and provided with a Plain Language Statement (PLS) with information about the qualitative interviews. In the second method of recruitment, DES frontline staff working with people with psychosocial disability provided potential participants with information about the qualitative study in the form of a flyer and PLS. DES frontline staff then assisted the lead author to make contact with interested potential participants.

Participants were asked to complete two semi-structured interviews with an interval of 6 months in between. Thirty baseline and 26 follow-up interviews were conducted with DES participants with psychosocial disability between November 2017 and April 2019. All interviews were conducted by the lead author. In the baseline interview, participants were asked about their life circumstances (family, education), mental health, work (including barriers, supports and aspirations); and, about their experiences with DES. In the second interview, participants were asked about any changes that had occurred in their lives since the first interview, including in relation to their life circumstances, mental health, and employment; supports received from DES provider and/or others; and, choice and control in their engagement with DES. Each interview lasted approximately 45 min. Interviews were audio-recorded and later transcribed.

### Qualitative narrative analysis

Narrative inquiry has been previously used to explore various lived experiences of phenomena (e.g. disability, supported-decision making, unemployment) [63–67]. Ridgway [68] for example used first person narratives to explore lived experiences of recovery of women with long-term psychosocial disability, describing how these narratives could inform recovery-orientated practice. Whilst a recent systematic review of mental health recovery reported on 45 separate studies documenting personal recovery narratives, demonstrating the wide utilisation of narrative inquiry to understand perspectives of recovery [69].

**Fig. 1:**
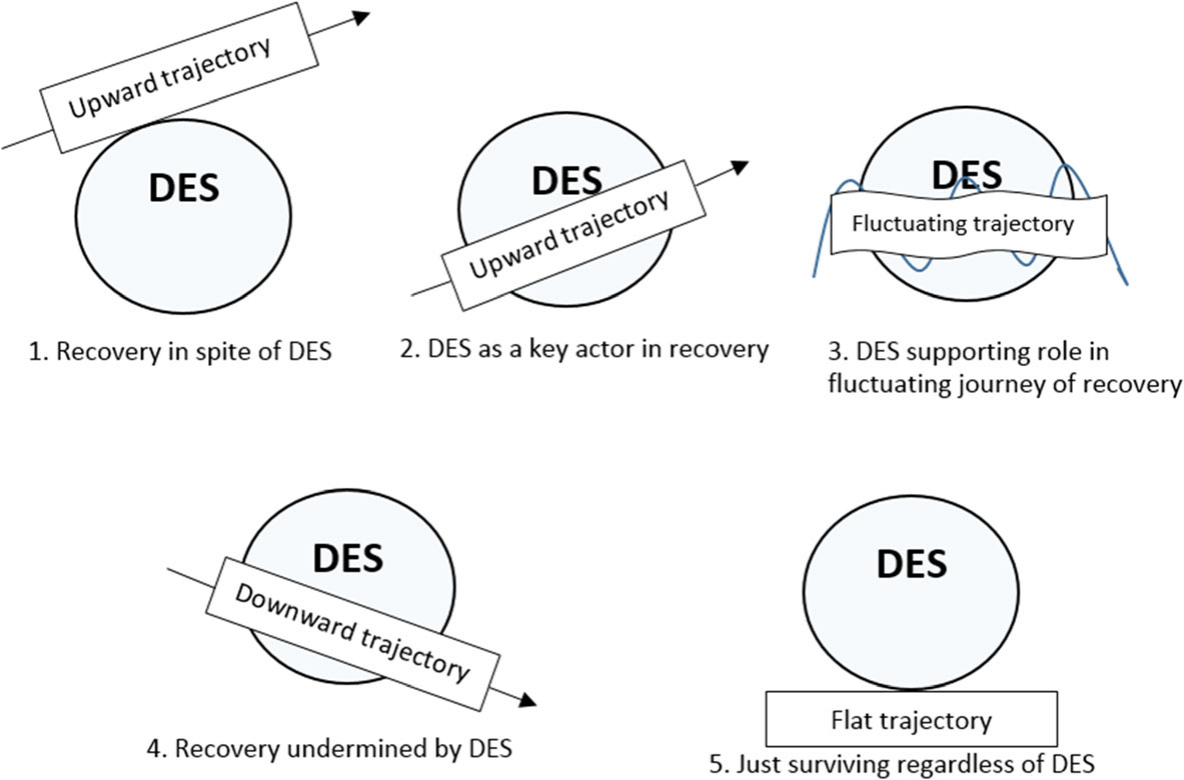
Visualisation of narrative positions

Our narrative analysis began with multiple readings of each transcript by the lead author. The initial focus was on identifying life circumstances (childhood, education, socio-economic conditions), experiences (mental health, work, DES), and relationships (family, DES and other services, within employment) given prominence by the interviewee and how these changed over time [63]. Data were then mapped to the components of the CHIME recovery framework. For example, if participants spoke about support (or lack thereof) with family or services, this data was grouped under Connectedness. Whereas data related to aspirations for work were grouped under Hope and optimism. Emerging themes that did not correlate well with the CHIME framework were grouped separately. For example, expectations of DES providers and whether and how expectations had been met formed a separate category outside of the a priori components of CHIME. Comparisons were then made across the cohort to identify similarities and differences in emerging narrative positions on recovery and whether and how DES was perceived as influencing these positions. For example, noting that recovery can be positioned in narratives as occurring within, despite of, or, outside of systems, some participants clearly talked about improvements in their mental health as occurring outside of the DES system. Whereas others clearly positioned improvements in mental health as occurring within DES. People may also follow different non-linear trajectories of recovery and see themselves at different stages of their journey (recovered, living well, making progress or surviving day-to-day) [69]. This was also evident in the emerging narratives, with some participants reflecting positive trajectories across all CHIME components, with others describing oscillating experiences. Draft findings were shared with the research team on an ongoing basis to ensure the emerging narrative positions were sensical and supported by the broader research and literature. Five main narrative positions were identified with each participant mapped to one of following narratives: 1) Recovery in spite of DES; 2) DES as a key actor in recovery; 3) DES playing a supporting role in fluctuating journeys of recovery; 4) Recovery undermined by DES; and, 5) Just surviving regardless of DES. See Fig. 1. below for a visual representation of the narratives. Logical.

## Results

### Demographics

The demographic characteristics of IDES respondents are presented in Table 1. Just under half of all respondents reported psychosocial disability as their main disability, with just under half of all other respondents reporting psychosocial disability as a co-occurring condition. IDES participants with psychosocial disability were slightly less likely to have a choice in which DES provider they accessed and were more likely to have been engaged with DES for longer when compared with participants with other disabilities. Qualitative participants were more likely to have finished secondary school when compared with IDES respondents, but less likely to have attained post-secondary qualifications. Compared with the current DES population, our sample has a higher proportion of females, and, people with psychosocial disability (48.0% vs 40.7%). There was a similar proportion of compulsory and voluntary participants between IDES respondents and current DES population, with more voluntary participants in the qualitative sample.

**Table 1:** Baseline demographics

IDES survey respondents					Qualitative respondents	Current DES population
		Psychosocial n(%)	Combined other n(%)	Total n(%)	n(%)	
Gender	Female	118 (66.7)	94 (49.0)	212 (57.5)	14 (46.7)	124,416 (46.8)
	Male	58 (32.8)	96 (50.0)	154 (41.7)	15 (50.0)	141,559 (53.2)
	Non-binary	1 (0.6)	2 (1.0)	3 (0.8)	1 (3.3)	NR
Age	18–24	21 (11.9)	24 (12.5)	45 (12.2)	1 (3.3)	39,084 (14.7)
	25–34	52 (29.4)	35 (18.2)	87 (23.6)	13 (43.3)	43,365 (16.3)
	35–49	60 (33.9)	46 (23.9)	106 (28.7)	13 (43.3)	73,490 (27.8)
	50 and over	44 (24.8)	84 (44.7)	126 (35.3)	3 (10.0)	109,736 (41.2)
Reporting other disabilities	Physical	38 (21.5)	15 (7.8)	53 (14.4)	6 (20.0)	NR
	Sensory	11 (6.2)	29 (15.1)	40 (10.8)	–	NR
	Psychosocial	–	88 (45.8)	94 (25.5)	–	NR
Highest level of schooling	< Primary school	2 (1.1)	1 (0.5)	3 (0.8)	–	NR
	Some high school <Y10	18 (10.2)	21 (11.0)	39 (10.6)	4 (13.3)	NR
	Year 10	33 (18.6)	46 (24.0)	79 (21.4)	1 (3.3)	NR
	Year 11	27 (15.3)	37 (19.3)	64 (17.3)	4 (13.3)	NR
	Year 12	96 (54.2)	86 (44.8)	182 (49.3)	21 (70.0)	NR
Post-school qualifications	None	32 (18.1)	48 (25.0)	80 (21.7)	16 (53.3)	NR
	Apprenticeship/ trade certificate	27 (15.3)	35 (18.2)	62 (16.8)	2 (6.7)	NR
	Other certificate (I-IV)	55 (31.1)	62 (32.3)	117 (31.7)	6 (20.0)	NR
	Associate degree/diploma	27 (15.3)	24 (12.5)	51 (13.8)	1 (3.3)	NR
	University degree	35 (19.8)	21 (11.0)	56 (15.2)	5 (16.7)	NR
Type of income support/ welfare benefit*	Newstart	131 (74.4)	142 (74.7)	273 (75.6)	19 (63.3)	202,059 (76.0)
	DSP	27 (15.34)	51 (26.6)	78 (21.2)	8 (26.7)	28,271 (10.6)
Engagement in DES	Compulsory	135 (76.3)	145 (76.7)	280 (76.5)	17 (56.7)	206,155 (77.5)
	Voluntary	42 (23.7)	47 (23.3)	89 (23.5)	13 (43.3)	59,820 (22.5)
Choice in provider	Choice	64 (48.9)	74 (51.7)	138 (50.4)	11 (36.7)	NR
	No choice	67 (51.1)	69 (48.3)	136 (49.6)	19 (63.3)	NR
Length of time with current DES provider	12 months of more	86 (53.4)	74 (43.5)	160 (48.8)	7 (23.3)	NR
Employment history	Ever in paid employment	160 (90.4)	173 (90.1)	333 (90.3)	26 (86.7)	NR
	Currently in paid employment	54 (33.7)	43 (24.9)	97 (29.1)	5 (16.7)	NR
Housing	Experienced insecure housing or no place to stay in last six months	25 (14.1)	18 (9.4)	43 (11.7)	8 (26.7)	20,126 (7.6)**
Total		**177 (48.0)**	**192 (52.0)**	**369 (100)**	**30 (100)**	**265,975 (100)**

*NR* Not reported by Department of Social Security (*DSS*) DSS only report primary disability which is described in the text

^*^ Welfare support in Australia includes various pensions and income support payments for people who are unemployed. Income support payments generally have mutual obligation requirements attached, i.e. recipients are obliged to actively look for work (compulsory job seeker status). Disability Support Pension sometimes have compulsory requirements depending on age and assessed level of capacity. Newstart is the main form of income support for Australians of working age who are unemployed. Two qualitative participants were not receiving any income support, while one was on sickness benefits

^**^DSS data reports homeless status as opposed to IDES which collected data on experiencing insecure housing or no place to stay in last six months

The majority of qualitative interview participants reported depression and/or anxiety as the main condition contributing to their disability, with others reporting psychosis and/or Post-Traumatic Stress Disorder. Many participants reported disrupted education, with a third not completing secondary school. Table 2. outlines the employment status of interview participants at baseline and follow-up, demonstrating the most common changes include moving from unemployment to studying, and, moving from part-time to full-time work.

**Table 2:** Employment status of qualitative interview participants at baseline and follow-up

Employment status	Baseline	Follow-up
Working full time	0	4
Working part time and requiring more hours	3	–
Working part time and studying	2	2
Studying and looking for work	1	4
Volunteering and looking for work	3	3
Volunteering and studying	1	2
Unemployed, not studying or volunteering	20	8
No longer in labour market or DES*	–	3

^*^One participant left DES and the labour market after becoming a parent

Two participants left DES and were not currently looking for work

### Participant expectations of DES workers

IDES respondents were asked a series of yes/no questions about what support they would like from their DES worker. Respondents with psychosocial disability were most likely to report they wanted their DES work to support them to feel confident in their abilities. This was followed by offering suggestions about what sort of work they may be suited to, and, wanting support once they were in a job. (See Table 3).

**Table 3:** Supports IDES respondents’ would like from DES workers

	Psychosocial n(%)	Combined other n(%)	Total n(%)
Support me to feel confident in my ability	114 (64.4)	109 (56.8)	223 (60.4)
Provide me with support when I have a job	104 (58.8)	125 (65.1)	229 (62.1)
Offer suggestions about what sort of work I might be good for	102 (57.6)	112 (58.3)	214 (58.0)
Help me apply for a job	96 (54.2)	103 (53.6)	199 (53.9)
Help me find a training course	89 (50.3)	86 (44.8)	175 (47.4)
Help me prepare for a job interview	84 (47.5)	86 (44.8)	170 (46.1)
Assist me with Centrelink	73 (41.2)	95 (49.5)	168 (45.5)
Help me participate in decision-making	59 (33.3)	67 (34.9)	126 (34.2)

### Mental health and well-being

As demonstrated in Table 4, IDES participants with psychosocial disability had on average significantly lower MHI-5 and PWI scores (means, 44.9 and 48.4, respectively) compared to participants with other disabilities (means, 55.8, 54.3). The PWI total scores for participants with psychosocial disability are below the normative score of 50 within the Australian population [60]. In terms of the PWI individual domains, participants with psychosocial disability report significantly lower levels of satisfaction across all domains compared with participants with other disabilities, with the exception of satisfaction with health.

**Table 4:** Comparison of mental health (MHI-5) and well-being (PWI) between individuals with psychosocial disability versus any other disability type combined. The seven PWI domain scores (scale between 0 and 10 or no satisfaction - complete satisfaction, respectively) are also presented. *P*-values are from linear regressions of disability type (psychosocial, other combined) on outcome variables (MHI-5 and PWI)

Outcome variables		Psychosocial mean (± 95% CI)	Combined other mean (± 95% CI)	p-value
MHI-5 total		44.9 (41.7, 48.2)	55.8 (52.2, 59.5)	< 0.01
PWI total		48.4 (45.3, 51.5)	54.3 (51.0, 57.5)	< 0.01
PWI individual domains – satisfaction with:	standard of living	5.5 (5.1, 5.9)	6.0 (5.6, 6.4)	0.07
	health	4.8 (4.4, 5.1)	4.4 (4.0, 4.8)	0.20
	achieving in life	3.9 (3.5, 4.3)	4.7 (4.3, 5.1)	0.01
	personal relationships	5.5 (5.2, 5.9)	6.1 (5.6, 6.5)	< 0.01
	how safe you feel	6.3 (5.8, 6.7)	7.0, (6.6, 7.4)	< 0.01
	feeling part of community	4.6 (4.1, 5.0)	5.2 (4.8, 5.6)	0.04
	future security	3.9 (3.5, 4.3)	4.7 (4.2, 5.1)	0.01

Linear regression analyses of survey respondents with psychosocial disability, adjusting for age, gender and education, found that while compulsory engagement with DES did not result in significantly lower PWI or MHI-5 scores when compared to voluntary participants, participants with psychosocial disability who reported having no choice in which DES provider they accessed, had significantly lower PWI and MHI-5 scores. We did not find evidence to show that length of time in DES influenced PWI or MHI-5 scores (See Table 4). Participants who were currently employed had higher PWI and MHI-5 scores compared with participants not currently in paid employment. Of participants with psychosocial disability who had ever worked, those currently working fewer or more hours than they would like had lower PWI and MHI-5 scores when compared to participants working about the number of hours per week that they would like. Individuals on permanent/fixed term contracts, also had significantly higher PWI and MHI-5 compared with individuals on casual contracts. In relation to choice in careers and mental health and well-being, survey participants who felt they had only some or no choice in their career options, had lower PWI and MHI-5 scores when compared to participants who felt they had complete choice over their career choices. Those reporting disability-related discrimination and housing insecurity were also more likely to have lower PWI and MHI-5 scores (Table 5).

**Table 5:** Factors affecting well-being and mental health for IDES respondents with psychosocial disability. *P*-values are from linear regressions of each exposure on PWI and MHI-5 outcomes and are adjusted for age, sex and education

Exposure variables		PWI total mean (± 95% CI)	p-value	MHI-5 mean (± 95% CI)	p-value
Completed secondary school	No	45.9 (38.6, 53.3)	–	41.5 (33.8, 49.2)	
	Yes	50.8 (37.0, 64.6)	0.14	44.0 (29.6, 58.5)	0.45
Compulsory engagement in DES	Yes	45.5 (38.1, 53.0)	–	40.9 (33.0 48.5)	–
	No	48.2 (33.1, 63.3)	0.49	45.6 (29.5, 61.5)	0.25
Choice in provider	Yes	52.4 (43.5, 61.2)	–	48.2 (38.8, 57.5)	–
	No	39.5 (23.8, 55.3)	< 0.01	36.1 (19.6, 52.5)	< 0.01
Length of time in DES	< 3 months	47.2 (36.1, 59.3)	–	42.5 (31.6 53.4)	–
	3–6 months	45.2 (21.4, 69.0)	0.76	38.0 (14.3, 61.7)	0.48
	6–12 months	46.3 (22.9, 69.8)	0.90	41.4 (20.8, 64.3)	0.85
	> 12 months	46.1 (25.8, 66.6)	0.84	40.8 (20.8, 60.8)	0.71
Experience disability-related discrimination	Yes	40.0 (32.7, 47.2)	–	34.7 (27.1 42.3)	–
	No	56.1 (42.8, 69.4)	< 0.01	51.2 (37.3, 65.0)	< 0.01
Ever employed	Yes	46.2 (38.6, 53.7)	–	41.4 (33.6, 49.2)	–
	No	44.8 (26.3, 63.3)	0.80	42.3 (22.9, 61.6)	0.90
Currently employed	Yes	53.2 (43.9, 62.5)	–	50.5 (40.8, 60.2)	–
	No	43.4 (27.0, 59.9)	0.01	39.9 (22.7, 57.2)	< 0.01
Preference to work	Fewer hours	41.8 (20.9, 62.7)	–	30.9 (10.7, 51.0)	–
	About the same	60.3 (20.5, 99.0)	0.05	56.3 (17.7, 94.9)	< 0.01
	More hours	53.7 (14.9, 91.7)	0.19	43.0 (5.2, 80.7)	0.13
Employment arrangement	Permanent or ongoing	65.8 (52.4, 79.0)	–	58.8 (44.6, 73.0)	–
	Casual or temporary	53.7 (29.0, 78.5)	0.04	43.7 (17.2, 70.3)	0.02
Choice in career/job	No choice	30.6 (21.0, 40.3)	–	34.1 (23.5, 44.7)	–
	Some choice	43.9 (26.0, 61.7)	< 0.01	40.5 (21.1, 29.4)	0.15
	Complete choice	54.3 (35.7, 72.8)	< 0.01	45.4 (25.1, 65.8)	0.02
Experienced no place to live/stay in last 6 months	Yes	38.5 (28.2, 48.8)	–	30.1 (19.2, 40.9)	–
	No	47.9 (28.7, 67.1)	0.03	43.9 (23.7, 64.1)	< 0.01

IDES respondents reporting choice in DES provider, were asked about the importance of various factors in informing this choice. Response options were scaled ‘Not important’, ‘Somewhat important’, and ‘Extremely important’ with Table 6 presenting the latter. Of most importance to all respondents was that the DES provider would recognise their strengths. This was followed by choosing a DES provider that was easy to get to, and, a provider that makes individuals welcome and has experience with their type of disability.

**Table 6:** Considerations when choosing a DES provider

	Psychosocial n(%)	Combined other n(%)	Total n(%)
Recognise my strengths	45 (72.6)	55 (76.4)	100 (74.6)
Easy to get to	49 (62.8)	51 (58.0)	100 (66.2)
Make me feel welcome	49 (62.8)	55 (62.5)	104 (62.7)
Experience with my disability	48 (61.5)	54 (61.4)	102 (61.5)
I will have the same consultant every time	45 (57.7)	52 (59.1)	97 (58.4)
Good reputation	47 (47.4)	37 (42.1)	74 (44.6)
Can use the internet	14 (18.0)	14 (15.9)	28 (16.9)

Note: Not all respondents responded to each item in the series

### Narratives of recovery

Similar to IDES survey respondents, interview participants’ often experienced poor mental health and well-being in and of itself, and, in relation to factors such as unemployment and/or the sense of control they felt in relation to their employment, insecure housing, and discrimination. Analysis of qualitative interview data enabled a more in-depth analysis of these factors, alongside exploration of the working relationship between DES workers and participants, leading to the formation of the five narratives.

#### Recovery in spite of DES

About one fifth of participants strongly resonated with a positive journey of recovery, attained in spite of their engagement with DES. Living with and learning from their experiences with mental health defined these journeys of recovery: ‘I feel like it has happened to me for a reason.’ (Participant#16 follow-up, P16b). Mental illness for these participants was often precipitated by trauma (military service, abuse from multiple perpetrators), with recovery facilitated by a combination of intrinsic traits (motivation, determination); support from others (family, peers); and, services (mental health, justice, rehabilitation).

*Two police officers saved my life … I was homeless at the time … I wanted to jump in front of a train. Not because I was depressed but because I completely wanted to go to heaven … They put their arm around me and they said they wanted to get an ambulance ‘Will you do that for us?’ … If they hadn’t stopped me that day I would be dead.* (P8 baseline, P8a)

Along their journey’s, participants had been receptive to supports and non-DES services they felt had been congruent with their needs and available at the right time: ‘You have to actually want help and be willing to utilise a service that you are getting to make it work probably.’ (P28b), yet acknowledged help is not always available: ‘It actually takes someone to hit rock bottom or even go to jail. It’s just so sad that it gets to that point.’ (P16b). Entwined in these concerns, was a pressure to stay well due to the limited availability of mental health services. There was also a strong desire to help others with mental illness and advocate for change and better systems of support.

*That is one thing that keeps me inspired to stay on top of things … I know how bad it can get. It would be good if we could try and get this stigma aside and help people get well. Because they are even closing down places, like mental health places.* (P8a)

*I want to live my optimum life and my optimum life is not about the outward things. It is about the optimal inside. I was not mad. People had corrupted my life … I am taking positive action to try and stop it … I am a real advocate. I am fighting for change.* (P24a)

Clearly articulated aspirations relating to work and the value of work were also central to these recovery journeys: ‘I want to be a community social worker, to help make the community better for him [newborn baby]. My main reason now, my main focus is to make everything better for my son.’ (P28b). For some, recovery was directly linked to attaining work within an organisational setting congruent with their skills, ways of working and values: ‘The culture is familiar and because the attitudes and the behaviours of the staff are similar to what I’ve had before, it is not like I’m having to completely reinvent myself to add value … this helped me be able to apply knowledge and skills and contribute. … So from that point of view there is less stress.’ (P4b). Others were not yet in careers they aspired to, but recognised their progression towards their ideal careers through paid work in other fields, volunteering or study: ‘I probably spent about two or three years recovering and now I’m back at Uni. Because I have got everything under control I’ve been able to get good grades … It can be stressful at times, but even with the volunteering, I come back home and I feel a lot better.’ (P14a).

Similar to the positioning of recovery occurring in spite of DES, attaining work and its role in supporting recovery, was defiantly spoken of as being achieved external to DES: ‘I did get this job on my own. It wasn’t like I had actually received a direct benefit from enrolling in the service.’ (P4b). Particularly during follow-up interviews, DES was often described as frustrating the process of finding work. People often felt their time was wasted by DES frontline workers who seemed unable to connect them with information, pathways or employers relevant to their job aspirations. Some people described being sent to inappropriate job interviews that left them feeling dejected, whilst others felt DES were not effectively responding to their complex employment barriers.

*Especially for someone that has got the disadvantage of a criminal record, and apparently they specialise in that? It’s such a long process and when you are wanting to work, it should be like ‘okay you want work, great’ and get you in the rhythm of getting into work rather than sitting in a funk … That’s a real struggle and that’s what I found … I got my own job so it’s too late now.* (P16b)

Not only was DES a hindrance for these participants in relation to finding work, they reflected DES should be more effective in supporting the recovery of people with mental health conditions: ‘Employment services need some counselling skills. They don’t have to be counsellors but they can help develop new ways for people to think.’ (P24a). Indeed, some participants who were not in work, felt their recovery was somewhat vulnerable within DES. ‘I’m proud of how far I’ve come. I’m just ready to work. I don’t want to not be in work for so long that I might start to go downhill internally. Because I might start to lose hope. So it may be like [DES provider] need to push a bit more.’ (P8b).

#### DES as a key actor in recovery

*My journey has been so hard for so long … I may have moments where I wish I had ended it, but at the end of the day I have a family that loves me … Years ago I wouldn’t have said that I want to get out there and be a productive member of society … It has been the support from my dad and mum and its helped big time. And it’s also thanks to* [DES worker] *because he has given me enough support.* (P30a)

Similar to narrative one, these participants took pride in their journeys of recovery but were more likely to acknowledge external support mechanisms facilitated their recovery. This was evident for approximately one quarter of participants. Finding meaning in their experiences similarly transpired into a desire to help peers: ‘I am really passionate about mental health … like getting people to connect … The majority of people who commit suicide, there is a lot of them that have been out of work for years. So I just feel that if people didn’t feel so alone and met other people in similar [circumstances] it might help’. (P12b). Hope and work-related aspirations were also present: ‘I want something more productive. I feel like before all I used to do was go to drop-ins and sit around and have coffee all day. I went to these groups for about 22 years. I feel I want something different now … I want volunteering or paid work.’ (P2b).

The main difference between this narrative and the first is the positioning of DES as a key component of their recovery: ‘[DES worker] has been like really helpful. Like all the way through. Like constantly checking up on me and he has been really engaged’ (P20b). Positive relationships with their DES worker were often described as a change for the better when compared to previous workers, and, emphasised as being proactive and effective: ‘The other job places … you signed in, stamp your name, leave. It seems they don’t really care about you. But here they actually talk to you and sit down and make plans with you and try to actually help … For the first time in a while I’m feeling good about getting a job.’ (P22a). Evidence of engaging with employers and job matching also featured more frequently in these narratives: ‘They said that they knew this place that was available, like looking for baristas. They put my name through and they gave me a call and wanted me to come in for a trial and yes, she was really happy.’ (P27b). Workers that were more readily able to identify and at least attempt to address multifaceted barriers to work were recognised as critical to employment outcomes and also demonstrated to participants that they were valued as a person.

[DES worker] *has been really concerned about getting me some kind of further help … just looking into different things that can help me or make me feel better physically and mentally … I think they are doing as best as they can until we come up with an idea and we try something or something falls into place.* (P19b)

There were, however, tensions in these stories. Some had been helped into full-time work - albeit not in something meeting their aspirations or desire for part-time work. This left people in a fragile position of being more stressed and too busy to access their mental health supports. They also found it difficult to access ongoing DES support to help with workplaces challenges and career development.

[DES worker] *said to stay in touch but I can’t get to them because I work when they’re open. Which is a pain because I love sitting down with* [DES worker] *and talking about everything. The other day I had a phone interview* [with DES] *but they never called and I was devastated because I could probably do with it … as much as I love this job I am always wondering what courses or things that I can do as well … and just to be able to debrief about little things that are upsetting me and get their advice … That is where I’m at with that and I try to cope. Keep strong … ride it out … I am not seeing the psychologist, because I don’t know where to fit this into my life anymore.* (P6b)

Similar to narrative one, some participants felt DES could be more effective if they were better integrated with mental health supports: ‘I think if there was a GP here or a nurse or a psychologist or social worker or something so when people are like that and they come in like that [experiencing significant mental health distress], they can talk to someone or a counsellor and I do know I think it would help.’ (P12a).

#### DES playing a supporting role in fluctuating journeys of recovery

This narrative was symbolised by fluctuating journeys of recovery punctuated by events and circumstances (family breakdown, homelessness, unemployment) that challenged participants’ mental health and sense of control over their lives: ‘It’s [suicide] tempting when you are in the depths of the darkness. But you always have to have some sort of hope and that is what the support group has got and the priest … very tiny steps that’s the way it is … whether I make it to the finish line in the end is another story.’ (P15a). The four participants within this narrative were typically tethered to their journeys by a sense of responsibility. This included caring for siblings due to parental mental illness or commitment to serve their community.

These participants were less socially connected compared with people in previous narratives: ‘I think probably years of being isolated most of the time … not necessarily written off but it is hard to find a group.’ (P20a). DES was therefore seen as a mechanism for social inclusion: ‘I was required to come in on a Monday but I used to come every couple of days to do the [online] course. It’s a nice place compared to the public library.’ (P20b). Work was also seen as important for improving their connections with others, with narrators unified by the positive belief that work was possible despite significant vocational and non-vocational barriers. DES was positioned as central to helping them achieve this, even if it was a long-term goal.

*It’s part of my process of trying to get my feet on the ground again. I want to serve, but I cannot compete on the same level as someone searching on* [job-seeking platform] *because of lack of experience. I have been out of the workforce for quite a while, plus maybe my mental illness may hinder this process. However, I thought to myself there must be an agency that caters for people like me … I gave a call to* [DES worker] *and she said come in.* (P15a)

Similar to the second narrative, positive relationships with their DES worker across the two interviews, was fundamental to helping them feel more empowered in relation to work: ‘He said to me “First up I’m not gonna get you a job, but I got the tools to help you get a job” … I did a mock interview last time when I was in this room. It wasn’t that great but when I did it again he said it was 1000 times better.’ (P23b). At the time of follow-up, all had been supported by their DES worker to engage with vocational training. DES workers were described as helping to identify courses aligning with their aspirations, covering costs, or encouraging participants when studying was challenging.

*There is actually some direction now. Even recently with* [DES provider] *I was like well I don’t really know what to do. So with everything I was reluctant. … Eventually we settled on something. Now everything is going forward and I am studying …* [Previous DES provider] *would be putting me in for jobs without asking me and it was just horrible. But here, it is like* [DES worker] *actually listens to me and he doesn’t do things without me, without making sure I’m okay with that.* (P29b)

Concerns were, however, raised during the second interview that services were changing, threatening the positive positioning of DES and creating anxiety. Internal training offered by DES for their clients, which also provided much valued peer support for example was less likely to be offered. There was also uncertainty regarding the stability of DES, precipitated by a higher than usual turnover of staff: ‘I’m just afraid that they might be leaving soon … they are changing people all the time … Are they going to close down? Because if they got a new provider, then somewhere down the line that place might go bust too.’ (P23b).

#### Recovery undermined by DES

Participants whose accounts aligned with this narrative were again proud of what they had overcome, accepting of their mental health, and, identified career paths where their experiences could help others. The four participants within this narrative were voluntary participants in DES and had strong aspirations to work.

*I won my life back. … I can look back and reflect and instead of regretting everything, I can say oh well I have learnt now. I can do my own research. Be my own boss. Don’t wait for people to do things for me … If I did a Diploma in Community Services I think I could get a job working with homeless people. I have firsthand experience and I think that would help … I just remember how hard that was … I was pregnant and scared … I do think it makes you a lot stronger when you come out of that*. (P25a)

Yet for these people, DES was distinctly positioned as undermining recovery with their initial optimism deteriorating over time. Whilst all experienced significant challenges in their lives (including housing and trauma related to previous violence), their engagement in DES and its impact on their mental health, recovery and employment outcomes dominated their stories: ‘I am really frustrated. At first my worker was like yes “this is my role, you have a disability you have a right to a reference so that you can get work again.” I think she rang them once or twice … and then she gave up, which is like writing off my career.’ (P1b).

Interviews highlighted these participants’ negative relationships with their DES workers whereby participants felt they were not listened to, and their skills and aspirations disregarded: ‘People need to listen to what they want and not just bulldoze over them. Which I feel that [DES provider] has been doing, like just bulldozing over me. Like telling me not to go for animal welfare jobs because there aren’t enough jobs out there. And when I go for [animal welfare] jobs, I see plenty.’ (P3b). DES workers also tended to allocate people into jobs that threatened their mental health. For some this pattern became intolerable and at the time of follow-up they had left DES and the labour market.

*I just found I kept going to* [DES provider] *but the girl was suggesting things that I understood she didn’t understand me and my illness. She would suggest to me to go and do traffic control on the road … my anxiety would be through the roof … they were not actually listening to me and thinking ‘let’s find a part-time job that will really suit her and that she can actually do’ … I just felt like it wasn’t going anywhere and it was wasting my time going there so I didn’t bother … I kept thinking I’m getting a bit unwell, I can’t do this anymore.* (P17b)

Concern was further raised that staff were not providing participants with supports they knew (or found out later) were available. This was incredibly distressing for participants as they already had so much to overcome when trying to find work. This knowledge, compounded by life circumstances, again led to people dropping out of DES. Through these examples, participants described approaches that they felt would benefit recovery as well as efforts to find work.

*I also found out there was ways they could have helped me after the situation had occurred … I was going to the pool to get ready [for an interview] … I was telling them about it, and she was saying ‘we have got a place here with a shower’ … I was informed of that two weeks before I left the service. … I didn’t have anything to wear for an interview … then I was told I could have had a voucher for clothing. That was really disappointing. I am sure they want to minimise the amount of resources they give to each client, but that would give them [participants] a better service … Looking for a job is quite a gruelling situation … It’s scary for a normal person let alone someone with mental health problems. Like those feelings of rejection are more intensified. I would have liked to have had an opportunity to debrief with them after each interview and maybe time with them before.* (P1b)

#### Just surviving regardless of DES

Narrative five was the most common with nearly a third of all participants mapped to this narrative. Their journeys were epitomised by a persistent, often exhausting battle of survival: ‘When you fight for so long you just get tired of it. So you don’t give up, you just give in.’ (P10b). Circumstances remained stagnant or deteriorated over time with few (if any) elements of recovery described. Mental health was a challenge in and of itself, but was often made more difficult by isolation, poor physical health and ongoing trauma: ‘I’m pretty sure many of the people they call me treatment resistant … I am still in the same overall situation. Being socially isolated is probably my biggest problem.’ (P21b).

Issues with DES and the labour market were woven into challenges encountered within broader systems (welfare, justice, education, health). Participants described themselves as slipping through systems: ‘I just slipped out of the [education] system somehow. It seems to be something I do very well.’ (P10a); finding systems difficult to negotiate: ‘I applied for the [disability] pension but I didn’t know how to go about it’ (P18a); or, having a sense of being under siege: ‘If I am to put my child first as I am, work is a secondary thing. And that is the whole point. The family law and the family violence process is designed to break people. … I will never have capacity to work again.’ (P9a). Participants also found it difficult to recognise their skills and how these could be harnessed to transition back into work: ‘I get overwhelmed, it’s especially even harder now, going through career change … when you’re feeling low you don’t feel like you have any [skills].’ (P26a).

Extensive periods of unemployment despite long-term engagement with DES were common. The few participants in this narrative that wanted to work felt let down by DES, often describing their DES workers as ineffectual and not understanding of their mental health, which made looking for work more challenging. Relationships with these workers had a negative impact on already precarious mental health as well as work aspirations. This was particularly so if they had a positive experience with a previous worker: ‘Before that I saw [previous worker] and he is fabulous … now I just feel like I’m coming in to hand in my list … It has really made me shut down over the last six months … now I feel stressed coming here.’ (P7b).

Others were engaged with DES more because of their welfare mutual obligations rather than thinking it would lead to employment outcomes. Relationships with DES and work in these instances differed across interviewees. For some, whilst the relationship with their DES worker remained positive, their mental health made it difficult to engage with DES and the labour market challenging: ‘I need to work but the mental health does take priority, it kind of strips it away. [DES] are doing a good job … there are still days when everything is just blank that is the best way to describe it. Half the time my world is empty, because it is easier being empty than full.’ (P10a). For others, the relationship with DES seemed mired in power imbalances, which subsequently seem to compound stigma related to unemployment: ‘There is pressure and guilt tripping involved … [DES worker] says “you know if you’re five minutes late we will dock your pay” [income support payment]. I think most people just turn up and keep quiet and make the right noises.’ (P21b).

## Discussion

Nearly half of our IDES survey respondents identified psychosocial disability as their primary condition, with just under half of all other respondents identifying co-occurring psychosocial difficulties [14]. These findings demonstrate a significant number of participants could benefit from recovery-oriented practices within DES. Our qualitative findings further highlight the positive influence on recovery that is possible when DES workers adopt approaches that align with recovery-orientated practice. Such approaches however were not consistently reported across the sample. Each narrative, alongside the quantitative findings, also reveal participants’ perspectives on how DES and the systems that surround it, could improve support towards the recovery and employment outcomes of people with psychosocial disability.

Consistent with government data highlighting Australians with disabilities experience poorer employment outcomes when compared to Australians without disability [5], just under a third of the IDES cohort reported they were currently employed. This is despite the majority of respondents being engaged with DES for at least 12 months. Respondents also reported a high prevalence of factors known to undermine access to work, including disability-related discrimination and insecure housing.

Re-iterating the important role work can play in recovery, quantitative respondents with psychosocial disability who were currently working, had significantly higher mental health and well-being scores compared to those not in paid employment. However it is possible that those with better mental health to begin with were more likely to be employed [11]. The positive relationship between work and mental health recovery was similarly observed in the qualitative findings. This is particularly so when participants found secure work that was congruent with their skills and aspirations, in organisations that aligned with their personal values and ways of working values, as seen in narrative one [70].

Individuals in narrative one were the most likely to be working, most strongly identified with all five elements of CHIME, and, were most strongly connected to the labour market. Their intrinsic motivation to work was often frustrated by the perceived ineffectiveness of DES. Indeed, those who were working reported employment was found independently of DES. Whilst DES was not positioned as supporting these journeys, these participants had been receptive to help provided through other non-DES services and supports. Participants highlighted the need for more investment in mental health services so people can receive support before they hit rock bottom. This was expressed alongside concern at the perceived decrease in availability of mental health services in Australia. Echoing previous research, concern was also raised regarding the limitations of DES frontline workers to adequately support participants, highlighting a need to develop basic skills in counselling and motivational interviewing [71].

Quantitative findings highlight that many respondents want DES providers to recognise their strengths and support them to feel more confident in their abilities to work. Qualitative participants aligning with narrative two and three seemed more receptive to the support they felt was provided to them within their engagement with DES. As highlighted in these narratives and other research, perhaps a greater individual need for support was matched with more highly-skilled workers that could work within DES to develop more positive worker-participant alliances [54, 72–74]. Such DES workers were described in ways that most resonated with recovery-orientated practice, in that they were reported to take the time to develop trusting relationships, and listen and respect the hopes and aspirations as well as the fears and concerns of participants. Whilst only a small number had been supported into work, the majority of participants within these narratives had been supported into further education and training. As observed in other studies, positive vocational steps towards work in turn helped these individuals on their journeys of recovery [36]. The importance of matching individuals with a DES worker that suits them is also evident in our findings. Individual DES workers were often reported to very effectively support the career aspirations and decision-making for some individuals and do so very ineffectively for others. DES participants are diverse, and building the capacity of DES workers to respond to diverse needs using recovery-orientated approaches would benefit more people [71]. Recovery-orientated approaches may similarly be encouraged by enabling participant choice in which DES worker they are attached to and to change if they feel positive support is not being achieved. Correspondingly and aligning with research by Simonsen et al. (2013), IDES respondents who felt they had complete choice in decisions relating to their careers and choice in DES provider, had higher mental health and well-being scores, when compared to those with only some or no choice.

As highlighted in this research and in the work of Milner et al. (2019) and Butterworth (2013), when the psychosocial needs of workers are undermined in work, the mental health and recovery of individuals is threatened. Providing post-placement support to individuals to help them manage psychosocial stresses in the workplace is thus vital. This was underscored by quantitative findings demonstrating the importance to participants of DES support when they do find work. Similarly, interview participants placed in full-time employment despite a desire for part-time work, found it hard to access their mental health supports. In these circumstances, ongoing employment support that is meant to be provided within DES was not being effectively implemented, placing some individuals in vulnerable positions in terms of their mental health and undermining their longer term career development [12]. This is supported by our quantitative findings, which highlight that IDES respondents with psychosocial disability who reported working ‘about the same hours’ as they would like had significantly higher mental-health and well-being scores when compared to people working more or fewer hours than they would like. Similarly, people with more secure working arrangements (permanent/fixed term) also had higher mental health and well-being than those who were casually employed. This resonates with research by Morgan et al. (2012) and Fasquilho et al. (2015) that highlight the challenge of finding secure employment in an era of casualization and precarious global economic conditions, and the negative impact this can have on mental health [5, 32, 75].

In stark contrast to the first three narratives, the experiences of individuals aligned with narrative four, highlight the negative impact on recovery and employment outcomes when positive working alliances are not achieved and when individuals do not feel listened to, with their hopes and aspirations disregarded. Our findings further align with research that finds mental health and employment outcomes are undermined when DES participants are pressured into jobs or training that don’t align with their skills or mental health needs, or, when participants perceive both inequities and ineffectiveness in the services they receive [76, 77].

Across the qualitative cohort, participants also described the stress associated with the high turn-over of staff occurring within DES, particularly since the 2018 DES reforms. Staff turn-over disrupts relational continuity and forces participants to re-tell their story and re-start the process of building trusting relationships with their workers [41, 44]. This process can take time and be re-traumatising for participants with psychosocial disability who often have difficulty with trust due to past experiences of harm or distress, including sexual assault or coercive interventions [78]. It also highlights the precarious environment DES providers operate within, the pressures on remaining DES staff and how this may impact on their capability to implement recovery-orientated approaches.

More than any other, narrative five underscores a need for greater investment in mental health supports for individuals experiencing significant psychosocial disability within and external to DES. These individuals were least likely to identify with any CHIME components of recovery and would clearly benefit from enhanced access to recovery-orientated community mental health services. This is alongside support to access key social determinants of mental health such as housing, which was a significant issue impacting on mental health and access to work for people across both the qualitative and quantitative cohorts. Yet as is so often emphasised by DES and mental health researchers, Australia must do much more to address systems level challenges to better integrate employment services with other mental health, rehabilitation and social services such as housing [2, 46, 50, 79].

### Methodological considerations

To our knowledge, this study represents the first survey of DES participants that incorporates existing validated surveys as well as supplementary items to comprehensively examine participant perspectives on factors that support and undermine access to sustainable and meaningful employment. However, the survey was implemented during a time when the DES sector was undergoing considerable reform which hindered recruitment. Because of the relatively small sample some of the estimates have wide confidence intervals due to insufficient power particularly when analyses were restricted to IDES respondents with psychosocial disability. Furthermore, items within the linear regression presented in Table 5, did not apply to all respondents with psychosocial disability, decreasing the response rate for particular exposure variables and restricting capacity to run multi-variate analysis. There is no available database for the DES population that can be used to recruit participants so participants were recruited through a relatively small number of DES partner services and email lists, meaning the sample may not be representative of DES participants. For example, our sample had a higher proportion of participants with psychosocial disability than reported in the DES population However, there were sufficient numbers of participants grouped under psychosocial disability and the combined other types of disability group to allow for comparisons between these group where relevant. Our sample did however have far fewer older participants compared with the broader DES population. Older Australians, with and without disability, are known to experience additional barriers to work on account of age-related discrimination [20]. Our findings may therefore not be representative of the disadvantage experienced by older DES participants. The survey was also only offered in English which is likely to have excluded people experiencing the intersectional effects of belonging to a Cultural and Language Diverse community and having a disability [14, 80]. Nonetheless, these findings represent an important contribution to the evidence on the relationship between employment and personal recovery and highlights critical factors for both future research and DES policy makers and providers to consider. Compared to the general DES population qualitative participants were more likely to be in receipt of the DSP and therefore have fewer mutual obligations to engage with DES. Similarly, our qualitative sample were more likely to be voluntarily engaged with DES when compared to the general DES population. These factors may indicate they have been assessed has having more significant disabilities (compared with the broader DES population) that impact on ability to work. They were also recruited through a relatively small number of DES providers and in essence self-selected into the study. Their experiences may therefore also not be representative of the broader DES population with psychosocial disability, or, have been exposed to other models of DES service delivery. The data clearly indicated five distinct narratives to which each qualitative participant could be mapped. However, we cannot rule out that a larger sample size may have identified further narrative positions, even though this is unlikely considering the clear alignment of all qualitative participants to one narrative position.

## Conclusions

Recovery-oriented approaches ensure services are delivered in a way that supports the personal mental health recovery of service users. Alongside access to secure and meaningful work, recovery was facilitated within the context of DES when frontline workers drew on these approaches to engage with participants. These approaches, however, were not consistently applied. Given the number of people with psychosocial disability moving through DES and the important role that work plays in supporting recovery, encouraging greater consideration of recovery-oriented practice within DES policy and programming and investment in building the capacity of frontline staff is warranted. Such efforts would be more effective however if systems-level barriers were simultaneously addressed. This includes an urgent need to improve access to recovery-orientated mental health services for people experiencing mental illness, together with greater integration and coordination between mental health and employment services to better support DES participants with psychosocial disability on their mental health recovery and employment journeys.

## Data Availability

The datasets generated and analysed during the current study are not publicly available as the research team did not seek permission from participants to share the data with third parties.

## Abbreviations

CATI: Computer-assisted Telephone Interview
DES: Disability Employment Services
IDES: Improving Disability Employment Study
MHI: Mental Health Index
NDA: National Disability Agreement
PWI: Personal Well-being Index
